# Syk phosphorylation – a gravisensitive step in macrophage signalling

**DOI:** 10.1186/s12964-015-0088-8

**Published:** 2015-02-03

**Authors:** Sonja Brungs, Waldemar Kolanus, Ruth Hemmersbach

**Affiliations:** Biomedical Research Institute of Aerospace Medicine, German Aerospace Center (DLR), Linder Hoehe, 51147 Koeln, Germany; Molecular Immunology, LIMES Institute, University of Bonn, Carl-Troll Str. 31, 53115 Bonn, Germany

**Keywords:** Host defence, Microgravity, Syk, Clinostat, NF-κB, Macrophage, ROS signalling

## Abstract

**Background:**

The recognition of pathogen patterns followed by the production of reactive oxygen species (ROS) during the oxidative burst is one of the major functions of macrophages. This process is the first line of defence and is crucial for the prevention of pathogen-associated diseases. There are indications that the immune system of astronauts is impaired during spaceflight, which could result in an increased susceptibility to infections. Several studies have indicated that the oxidative burst of macrophages is highly impaired after spaceflight, but the underlying mechanism remained to be elucidated. Here, we investigated the characteristics of reactive oxygen species production during the oxidative burst after pathogen pattern recognition in simulated microgravity by using a fast-rotating Clinostat to mimic the condition of microgravity. Furthermore, spleen tyrosine kinase (Syk) phosphorylation, which is required for ROS production, and the translocation of the nuclear factor kappa-light-chain-enhancer of activated B cells (NF-κB) to the nucleus were monitored to elucidate the influence of altered gravity on macrophage signalling.

**Results:**

Simulated microgravity leads to significantly diminished ROS production in macrophages upon zymosan, curdlan and lipopolysaccharide stimulation. To address the signalling mechanisms involved, Syk phosphorylation was examined, revealing significantly reduced phosphorylation in simulated microgravity compared to normal gravity (1 *g*) conditions. In contrast, a later signalling step, the translocation of NF-κB to the nucleus, demonstrated no gravity-dependent alterations.

**Conclusions:**

The results obtained in simulated microgravity show that ROS production in macrophages is a highly gravisensitive process, caused by a diminished Syk phosphorylation. In contrast, NF-κB signalling remains consistent in simulated microgravity. This difference reveals that early signalling steps, such as Syk phosphorylation, are affected by microgravity, whereas the lack of effects in later steps might indicate adaptation processes. Taken together, this study clearly demonstrates that macrophages display impaired signalling upon pattern recognition when exposed to simulated microgravity conditions, which if verified in real microgravity this may be one reason why astronauts display higher susceptibility to infections.

## Background

Astronauts suffer from various infectious diseases such as respiratory and urinary tract infections and a higher susceptibility to the reactivation of persistent viruses, e.g. Epstein-Barr virus [1]. In addition, pathogens display increased virulence when exposed to a microgravity environment [2]. As a consequence, the feasibility of astronauts suffering from infections due to an impaired immune system and/or the increased virulence of microbes onboard the International Space Station (ISS) [3] might be increased.

Cells of the innate immune system are the first line of immunological defence and are capable of killing invading pathogens. Cells of the myeloid lineage respond mainly to the presence of pathogenic patterns like fungi, bacteria and viruses as well as dead host cells, by recognising and removing them. The pathogen-associated molecular patterns (PAMPs) of microbes are recognised by receptors on the cell surface of neutrophils and macrophages (for a review, see Akira et al. [4]). For example, zymosan, a fungal and yeast cell wall component consisting of 1,3 β-glucan linked to chitin and 1,6 β -glucans [5], is recognised by Toll-like receptor 2/6, Dectin-1 and complement receptor CR1/3 [6]. Furthermore, zymosan can be opsonized with antibodies and complement factors and thus is additionally recognised by Fcγ cell surface receptors. After recognition at the cell surface, the particle is internalised into the cell and nicotinamide adenine dinucleotide phosphate (NADPH) oxidase, a multi-subunit enzyme, is assembled to produce the radical superoxide within the phagosome to digest the engulfed pathogen [7-9]. This process is called the oxidative burst and involves different radical types which are generated by several enzymatic processes [9].

The 72 kDa spleen tyrosine kinase Syk is activated by the adapter proteins of pattern recognition receptors (PRR) on the cell surface by Src kinase and/or the immunoreceptor tyrosine-based activation motif (ITAM) and plays a crucial role in different biological functions [10]. Syk triggers ROS production upon PRR stimulation by a so far unknown mechanism [10]. Underhill et al. [11] showed that zymosan is phagocytosed normally in Syk-deficient mice but ROS production is abolished. Furthermore, Syk plays an important role in the activation of transcription factors like NF-κB [12] and nuclear factor of activated T cells (NFAT) [13]. The activation of transcription factors modulates the immune response, leading to inflammation, which is a crucial feature of macrophages. After recognition, engulfment and the oxidative burst, the transcription factor NF-κB becomes activated to initiate downstream signalling and inflammatory response. Tripathi and Aggarwal [14] describe how the activation of this transcription factor takes place. The IκB subunits inhibit the translocation of NF-κB to the nucleus and become phosphorylated upon Dectin-1 receptor and Toll-like receptor (TLR) stimulation. Degradation of IκB is accomplished by oxygen radicals as well as phosphorylation by Syk kinase [15]. After NF-κB translocation to the nucleus, the transcription of a variety of genes is initiated. Cytokines, chemokines and adhesion molecules are expressed, which are crucial for recruiting neutrophils and modulating inflammation and the adaptive immune response [16].

Cells of the myeloid lineage play a critical role in the first line of defence of the innate immune system. So far, several studies have pointed out the high sensitivity of this cell type to microgravity, i.e. neutrophils and monocytes of astronauts after spaceflight display a reduced capability to phagocytose pathogens and to produce reactive oxygen species during the oxidative burst [17,18]. This was assumed to be due to stress related to the duration of spaceflight. Adrian [19], Horn [20] and Huber [21] were able to show that macrophages respond to real and simulated microgravity with a reduction in ROS after stimulation with the yeast cell wall component zymosan. Rat macrophages increased their ROS production after zymosan stimulation in hypergravity (1.8 *g*) as shown during parabolic flight and centrifugation [19]. However, the underlying mechanism remained to be elucidated. It is not clear how gravity influences the crucial features of macrophages that lead to the establishment of host defence. It can be assumed that a failure in phagocytosis as well as the oxidative burst of macrophages plays a critical role in the immune deficiencies observed in astronauts.

This study was conducted to provide further insight into the effects of altered gravity on the oxidative burst in macrophages. It complements our earlier study [19] by focusing on the identification of the underlying signalling pathways. Clinorotation, that means fast rotation of a sample around an axis perpendicular to the force of gravity, an established method in the European Space Agency (ESA) ground-based facility program [22], was used to expose cells to simulated microgravity conditions. It has been shown that clinorotation for our chosen test system mimics the microgravity environment very well, and can be used as a simulator to study the impact of microgravity (see review of Herranz et al. [19,22]).

Here, we further characterise ROS production in NR8383 rat macrophages and show the influence of simulated microgravity on Syk phosphorylation and NF-κB activation upon pattern recognition. We identify Syk phosphorylation as a gravisensitive step within the ROS signalling pathway.

## Materials and methods

### Cell cultivation

The rat macrophage cell line NR8383 (a gift from the Karlsruhe Institute of Technology) was cultivated at 37°C with 5% CO_2_ and 95% humidity in HAMS F12 medium (Biochrom), supplemented with 10% foetal calf serum (FCS) (Biochrom), 1% penicillin-streptomycin (Biochrom) and 0.1% 2-mercaptoethanol (Merck).

For electric mobility shift assay (EMSA) experiments, cells were cultivated in very low endotoxin (VLE) DMEM (PAA) with 2% FCS (Biochrom) to diminish endotoxin contamination.

### Luminol assay

To determine the production of reactive oxygen species, the Luminol assay was utilised. This assay was adapted to Allen [23] and is analogous to the protocol of Horn [20] and Huber [21]. Luminol is a cell-permeable substance which detects radicals inside as well as outside of a cell. In the presence of an oxidiser, in this case oxygen ions and peroxides which are generated during the oxidative burst, Luminol becomes oxidised. This process can be catalysed by the addition of the horseradish peroxidase (HRP). During this reaction, Luminol undergoes a conformation change which in turn leads to the emission of light. The radical production of the cells oxidises the Luminol and a conformational change of the molecule leads to the emission of light, which is measured by a photomultiplier tube (PMT) connected to a frequency counter (BK Precision) and a laptop (Fujitsu Siemens). A 1 ml cuvette which was subjected to clinorotation at 60 rpm (see section PMT-Clinostat) was filled with 792 μl of the cell suspension at a concentration of 7×10^5^ cells/ml, 165 μl of Luminol (10 mM, AppliChem) in borate buffer (0.2 M H_3_BO_4_, 0.02 M Na_2_B_4_O_7_×10H_2_O, pH 9 [both Roth]) (from a 100 mM stock solution of Luminol in DMSO), 33 μl of horseradish peroxidase (Merck) in Phosphate Buffered Saline (PBS) (500 U/ml) and 10 μl of a stimulating substance, i.e. zymosan from *Saccharomyces cerevisiae* (Sigma) (100 μg in PBS), lipopolysaccharide (Sigma) (100 ng in PBS) or curdlan from *Alcaligenes faecalis* (Wako chemicals) (500 μg in 0.1 M NaOH [Roth]). Each experiment was performed for 45 min.

### Western blot analysis

Samples were prepared from the Pipette-Clinostat. Up to 6×10^6^ cells were stimulated with 10 μg/ml opsonized or non-opsonized zymosan and exposed to clinorotation. Opsonized zymosan was prepared according to the protocol of Allen, Huber and Horn [19,21,23]. Non-stimulated cells (treated with PBS instead of zymosan) were run as a control. After 15 min, samples were directly transferred to ice-cold PBS, centrifuged (300 *g* for 5 min), washed with ice-cold PBS and proteins were extracted with 8 M urea extraction buffer (Roth), supplemented with PhosphoSTOP and ProteinaseComplete (both from Roche), according to the supplier’s instructions.

Determination of the amount of protein was done according to the standard bicinchoninic acid assay (BCA) protocol (BioRad). 40 μg protein samples were boiled with loading buffer (0.28 M Tris–HCl pH 8, 30% glycerol, 10% sodium dodecyl sulphate (SDS), 60 mM DTT, 0.0012% bromphenol blue) at 99°C for 5 min. After centrifugation for 5 min at 11,300 *g*, samples were separated on a 10% SDS gel (approx. 90 min, 120 V) and transferred to a nitrocellulose membrane (PROTRAN Schleicher & Schuell) for Western blotting (120 min, 80 V). The membrane was blocked with 5% milk powder (Roth) and then incubated with anti phospho-Syk (Tyr525/526) rabbit antibody (Cell Signalling) 1:500 in Tris-buffered saline tween-20 (TBST) (0.5 M Tris–HCl, pH 7.5; 1.5 M NaCl and 0.06% Tween) overnight at 4°C with gentle shaking. Sample loading controls were detected by using a rabbit β-actin antibody (1:1000; Sigma) in 5% milk powder in TBST. After three washes in TBST, the secondary goat anti-rabbit antibody IRDye800DW (Li-cor) was applied at 1:5000 for 1 h at room temperature in 5% milk powder in TBST. Band detection was performed with the Li-cor Odyssey imager.

We collected each unstimulated, opsonized Zymosan and non-opsonized Zymosan stimulated samples of 1 *g* controls (n = 5) and μ*g* (simulated microgravity) (n = 5) samples for Western blot analyses (n = 5).

### EMSA

3×10^6^ cells were stimulated with approximately 625 μg/ml of opsonized zymosan for 4 h during clinorotation on the Pipette-Clinostat. Samples were collected and stored on ice.

Nuclear extracts were prepared in a buffer containing 10 mM HEPES, 10 mM KCl, 0.1 mM EDTA, 0.1 mM EGTA, 1 mM DTT and 1 mM PMSF. 25 μl of 10% Igepal was added after 15 min and incubated for 1 min at 4°C. Nuclei were pelleted by centrifugation at 11,300 *g* at 4°C for 5 min and resuspended in 50–70 μl of buffer containing 20 mM HEPES, pH 7.9, 0.4 M NaCl, 1 mM EDTA, 1 mM EGTA and 1 mM DTT. The nuclear envelope was pelleted by centrifugation at 11,300 *g* for 5 min at 4°C. The nuclear extracts (supernatants) were collected and stored at −80°C. The protein concentration was determined by a standard BCA-assay (BioRad).

20 μg of NF-κB sense and antisense strand (Eurofins MWG Operon) were annealed and then labelled with 12 μl of gamma-^32^P-ATP (Perkin Elmer) in kinase buffer. Firstly, 10 μg of the nuclear extract was incubated for 20 min at 4°C with 2 μl of poly dl:dC (1 μg/μl) to reduce non-specific NF-κB protein binding. Secondly, nuclear extracts were incubated with 2 μg of a radioactively labelled NF-κB recognition sequence in a binding buffer (25 mM HEPES (pH 7.8), 25 mM MgCl_2_, 250 mM KCl, 1 mM EDTA, 50% glycerol and 25 mM DTT) in a nuclear protein-DNA binding reaction for 7 min at room temperature.

Samples were incubated with loading buffer and run on a non-denaturing gel at 200 V for 2–3 h. After drying the gel (2 h, 80°C under vacuum) it was exposed to radiographic film at −80°C for 24–96 h.

We collected each unstimulated and opsonized Zymosan stimulated samples of 1 *g* controls (n = 4) and μ*g* (simulated microgravity) (n = 4) samples from Pipette-Clinostat for EMSA analyses (n = 4).

### 2D Clinostat systems for microgravity simulation

#### PMT-Clinostat

**Figure 1:**
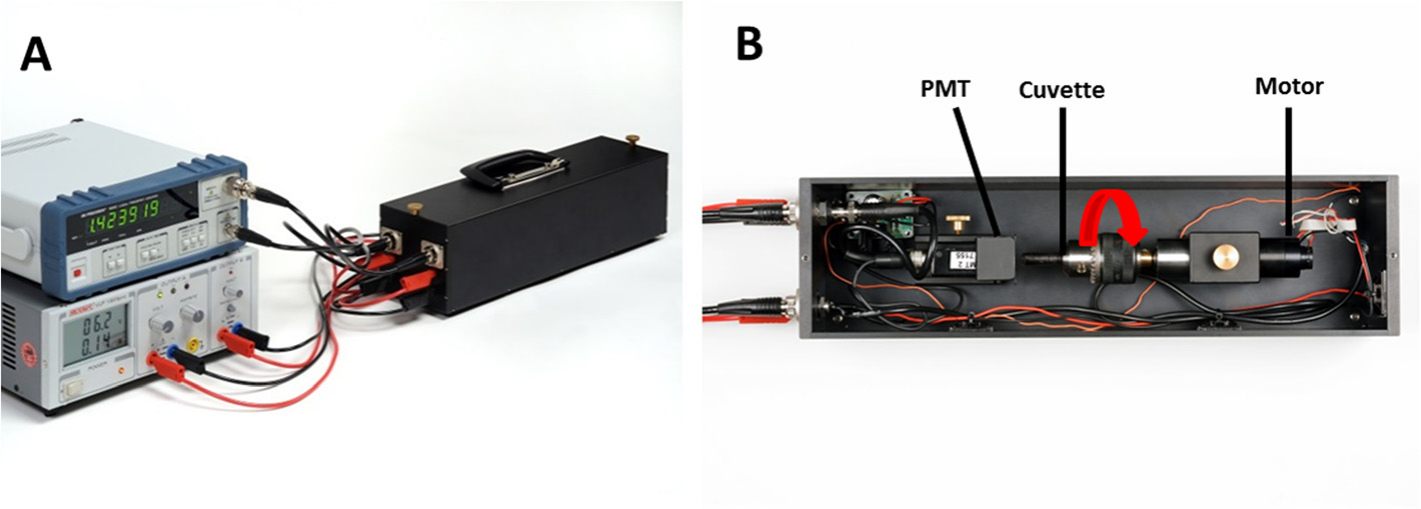
**PMT-Clinostat used in this study.** PMT-Clinostat **(A)** according to Horn et al. [24] and Adrian et al. [19], **(B)** top view of the PMT-Clinostat. The red arrow indicates the horizontal rotation axis of the sample cuvette.

The photomultiplier (PMT)-Clinostat (Figure 1A) published by Horn et al. [24] enables bioluminescent measurements under fast clinorotation. Here, it was used to determine reactive oxygen species production under clinorotation (simulated microgravity conditions). Clinorotation means fast and constant rotation of the samples around an axis perpendicular to the force of gravity, leading to cancellation of the gravity influence. In the chosen experimental approach, the sample diameter was kept small with respect to the quality of the simulation: at the chosen parameters, the sample cuvette with a diameter of 4 mm was rotated at 60 rpm resulting in a maximal residual acceleration, corresponding to ≤ 0.008 *g*. The cuvette containing the cells was combined with a photomultiplier tube in front (Figure 1B), in order to amplify the light signal of the cells, which was recorded by a frequency counter connected to a computer. All experiments were carried out in an incubator at 37°C. 1 *g* controls were prepared under the same conditions, but without rotation of the cuvette.

PMT-Clinostat experiments (1 *g* and simulated microgravity) had to be performed consecutively, since only one sample could be measured at a time. After 45 min of measurement, the sample was changed and a new experiment was started. Due to measurements on different days, the data was normalised.

### Pipette-Clinostat

**Figure 2:**
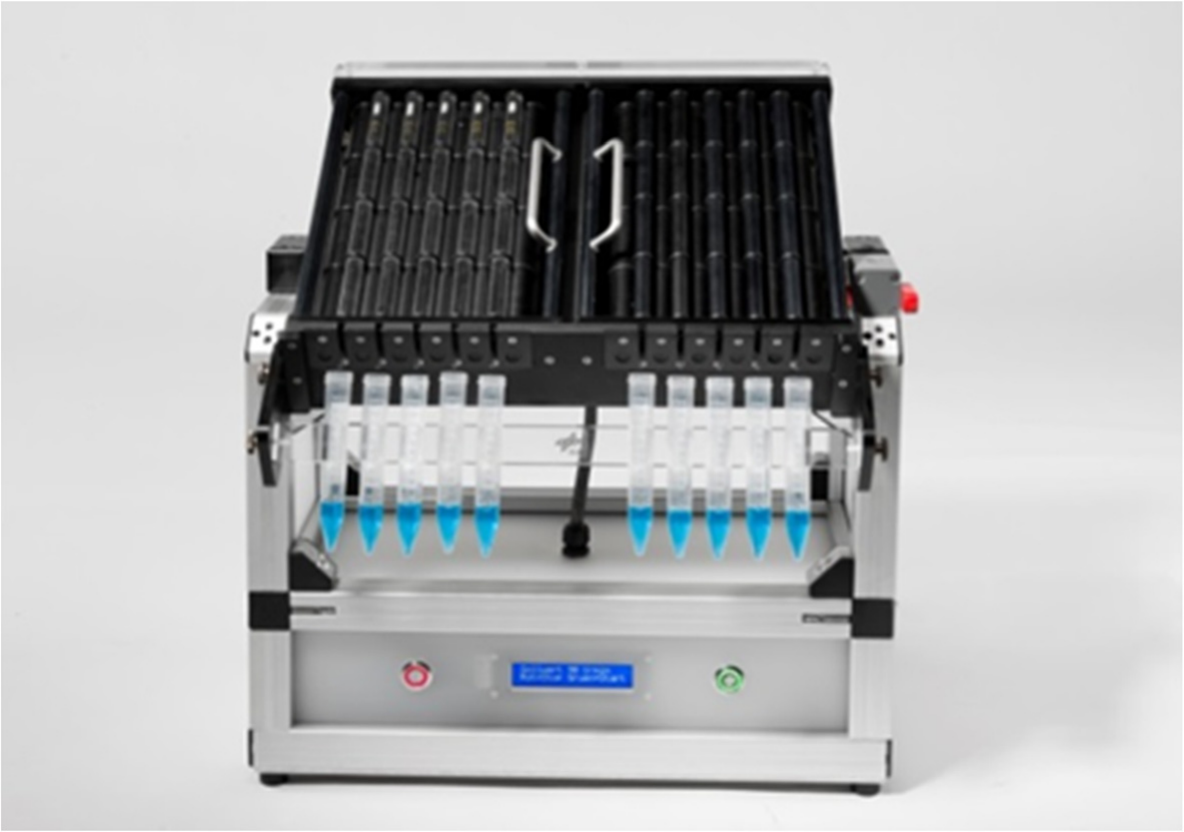
**Pipette-Clinostat developed at DLR (Design: Jens Hauslage).**

The same Pipette-Clinostat (Figure 2) as used in the studies by Horn [20] and Adrian et al. [19], consisting of ten 1 ml pipettes (diameter 3 mm) rotated along their horizontal axis at 60 rpm, was used in the present study. Pipettes were filled with 500 μl of the cell suspension and directly transferred to the Clinostat, or served as a static 1 *g* control. Fixation was performed during clinorotation (rotating pipette) or under 1 *g* conditions (static pipette; control) followed by Western blot and EMSA analysis. Under the chosen Clinostat set-up, the residual acceleration acting on the cells was less than ≤ 0.006 *g*. All experiments were carried out in an incubator at 37°C. 1 *g* controls were prepared under the same conditions, but without rotation of the pipettes.

### Statistics

Data of the PMT-Clinostat experiments were normalised due to collection on different days. After a Grubbs outlier test, a Kolmogorov-Smirnov test for normality was carried out. The level of significance was estimated with Student’s t-test or ANOVA (Tukey post hoc test) with IBM SPSS statistics 19. Differences were considered significant at the level of p < 0.05. All data are represented as mean ± standard deviation. *p < 0.05; **p < 0.01 and ***p <0.001.

## Results and discussion

### ROS production is reduced under simulated microgravity conditions (clinorotation) upon stimulation by non-opsonized zymosan

In the study by Adrian et al. [19], we extensively characterised the oxidative burst after stimulation with opsonized zymosan particles, showing that both real and simulated microgravity as well as hypergravity have an impact on the ROS production, showing either a decrease (microgravity) or an increase (hypergravity) compared to the 1 *g* control. Furthermore, we verified our chosen simulation approach (the Clinostat method) under real microgravity conditions during parabolic flight [19]. Thus, we can assume that clinorotation of macrophages effectively mimics the conditions that can be expected in real microgravity.

**Figure 3:**
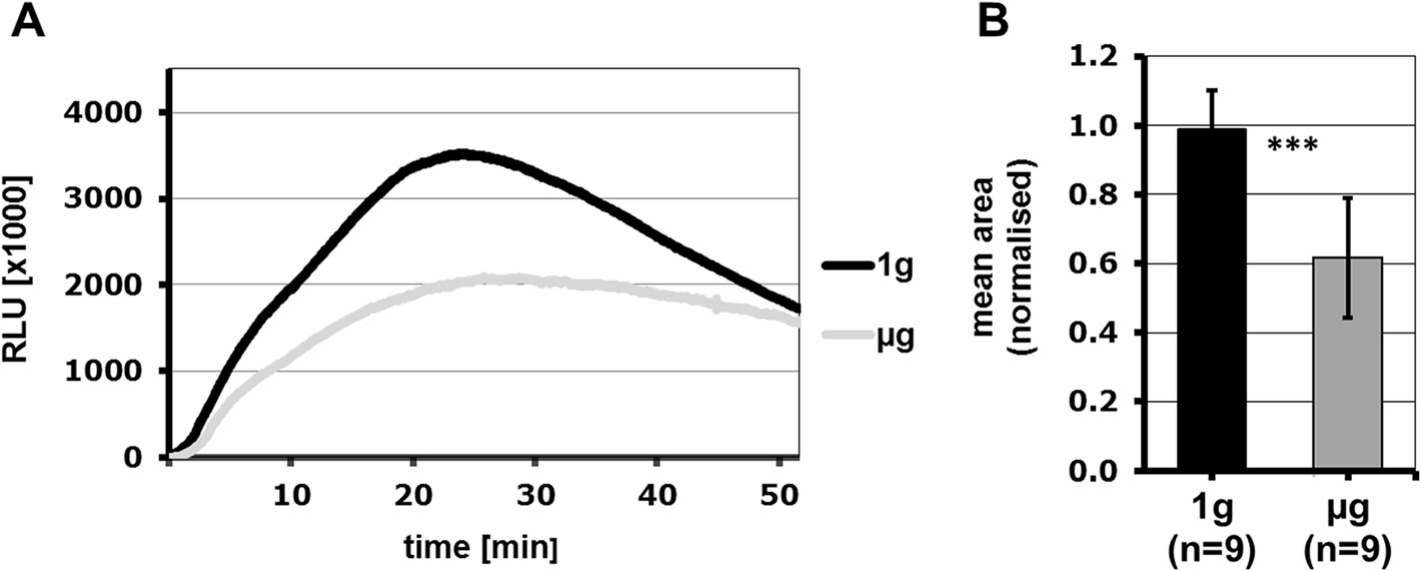
**ROS production is significantly reduced in simulated microgravity when TLR2/6, Dectin-1 and complement receptors are stimulated by non-opsonized zymosan.** The influence of simulated microgravity (PMT-Clinostat) on the ROS production of the cell line NR8383 after stimulation with 100 μg/ml non-opsonized zymosan, determined by the oxidation of Luminol **(A)** and calculated as total ROS production (area under the curve) **(B)**. Normalised data are shown with standard deviation. Significance was estimated by means of Student’s t-test (***p < 0.001).

Opsonized zymosan is recognised by a variety of cell surface receptors such as TLR 2/6, CR and Dectin-1 and Fcγ receptors. Therefore, NR8383 macrophages were stimulated with 100 μg/ml non-opsonized zymosan, and ROS production was monitored under simulated microgravity conditions. Figure 3 shows that ROS production was significantly reduced if TLR 2/6, Dectin-1 and complement receptors were stimulated by non-opsonized zymosan compared to the static 1 *g* control. The highly significant reduction in the production of oxygen radicals in simulated microgravity using non-opsonized zymosan is comparable to the results of Adrian et al. [19] showing the effects of simulated and real microgravity on ROS production after opsonized zymosan stimulation.

### Stimulation with lipopolysaccharide (LPS) and curdlan leads to diminished ROS production in simulated microgravity, indicating the gravisensitivity of distinct signalling pathways

**Figure 4:**
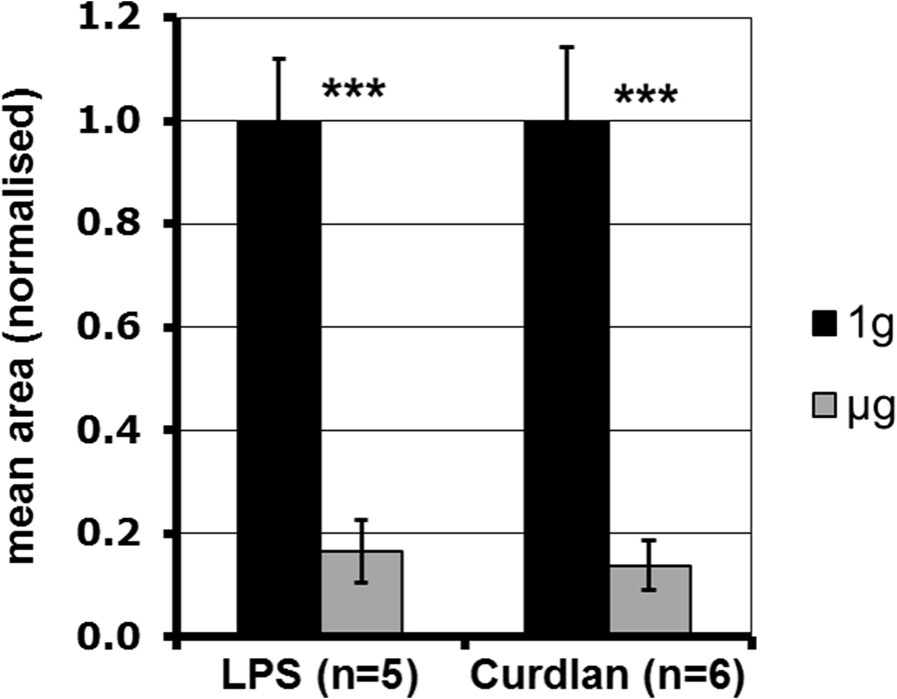
**Simulated microgravity reduces ROS production after the stimulation of TLR 4 by LPS, or Dectin-1 by curdlan, respectively.** The influence of simulated microgravity (PMT-Clinostat) on ROS production (area under the curve) after stimulation with curdlan (500 μg/ml) or LPS (100 ng/ml) for 50 min, determined by the oxidation of Luminol. Normalised data are shown with the standard deviation. Significance was estimated with Student’s t-test (***p < 0.001).

Individual pattern recognition pathways were stimulated by using LPS and curdlan, which are recognised by TLR and Dectin-1, respectively. Both substances led to reduced ROS production in simulated microgravity (Figure 4) compared to 1 *g*, comparable to the results using either opsonized [19] or non-opsonized zymosan (Figure 3). In summary, we can state that distinct pathways are sensitive to simulated microgravity and that the impaired oxidative burst in microgravity is not dependent on a single pathway.

As previously shown by Adrian et al. [19], phagocytosis after opsonized zymosan stimulation is reduced in microgravity. Since the effects on ROS production are stronger than the reduction in phagocytosis, a direct effect on signalling has been proposed, supported by the current study revealing a significant reduction in the ROS level after stimulation with non-opsonized zymosan as well as LPS and curdlan. Furthermore, changes in ROS production occur rapidly (within seconds) and reversibly in response to altered gravity, as shown during parabolic flight studies [19], while phagocytosis requires more time. We propose that the strong impact of microgravity on the ROS production capability of macrophages arises from impaired signalling. The pattern recognition receptor pathways leading to ROS production have several steps in common. Since the changes in ROS production according to altered gravity occur in a very fast manner, we assume that fast signalling steps close to the cell surface are altered under different gravity conditions. The spleen tyrosine kinase Syk plays an important role in ROS production following stimulation of the cell surface receptors Dectin-1 [11], CR [25], Fcy [11,25,26], TLR2/6 and TLR4 [27,28]. Recent studies have shown that Syk plays an important role in TLR 4 signalling: the Syk inhibitor piceatannol abolished ROS production in LPS stimulated neutrophils [29]. Since Syk is involved in each of the pathways investigated in this study, leading to the production of ROS, we decided to further investigate Syk under simulated microgravity conditions.

### Impact of simulated microgravity on ROS signalling: impaired Syk phosphorylation

The signalling pathway leading to ROS production after cell surface receptor stimulation requires the phosphorylation of the spleen tyrosine kinase Syk, which is required for ROS production and its involvement in the proximal signal transduction of important immuno-receptors has been shown by several investigators [10,11]. The treatment with the Syk inhibitor piceatannol leads to a completely abolished ROS production after stimulation with zymosan or curdlan (Dectin-1 receptor) stimulation [11]. Furthermore, Dectin-1 and TLR4 collaborate through the Syk pathway [30] emphasising the importance of Syk. The phosphorylated tyrosines (Tyr 525/526) in the activation loop of Syk are a good indicator for Syk function [31], thus, the phosphorylation of the kinase enzymatic site at Tyr525/526 was investigated and analysed by Western blot.

**Figure 5:**
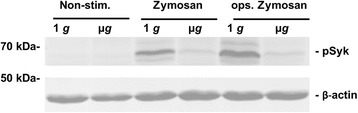
**Phosphorylation of Syk is impaired in simulated microgravity (Pipette-Clinostat).** Western blot analysis of phosphorylated Syk protein (Tyr525/526) in NR8383 cells stimulated with 100 μg/ml non-opsonized zymosan and opsonized zymosan, respectively, in simulated microgravity (μ*g*) and 1 *g* controls for 15 min. β-actin was used as a loading control. The blot represents one out of 5 individual experiments.

Cells stimulated with either non-opsonized zymosan or opsonized zymosan were exposed to 15 min of clinorotation in the Pipette-Clinostat. Figure 5 shows one representative Western blot of several experiments (n = 5). Phospho-Syk was nearly abrogated in the simulated microgravity samples after zymosan stimulation, independent of its opsonization status. This impairment clearly shows that the Syk kinase phosphorylation is a gravisensitive process which leads to a reduction in ROS production.

Nevertheless, other steps in the signalling cascade could also be impaired by simulated microgravity, for example NADPH oxidase itself, which is a multi-subunit enzyme. Impaired assembly of the subunits might also be influenced by the lack of gravity.

ROS and activated Syk lead to the phosphorylation and then degradation of NF-κB inhibitory protein IκBα. After phosphorylation and ubiquitination of IκBα, the NF-κB complex p65/p50 is in its active conformation and is translocated to the nucleus. The transcription factor NF-κB promotes the production of pro-inflammatory cytokines and chemokines and is very important in immune responses and in activation of the adaptive immune system [14]. The lack of effective Syk phosphorylation and ROS production may lead to abrogated activation of the transcription factor NF-κB. Therefore, we investigated NF-κB translocation to the nucleus during 4 h of simulated microgravity.

### NF-κB activation is not influenced by simulated microgravity upon opsonized zymosan stimulation

**Figure 6:**
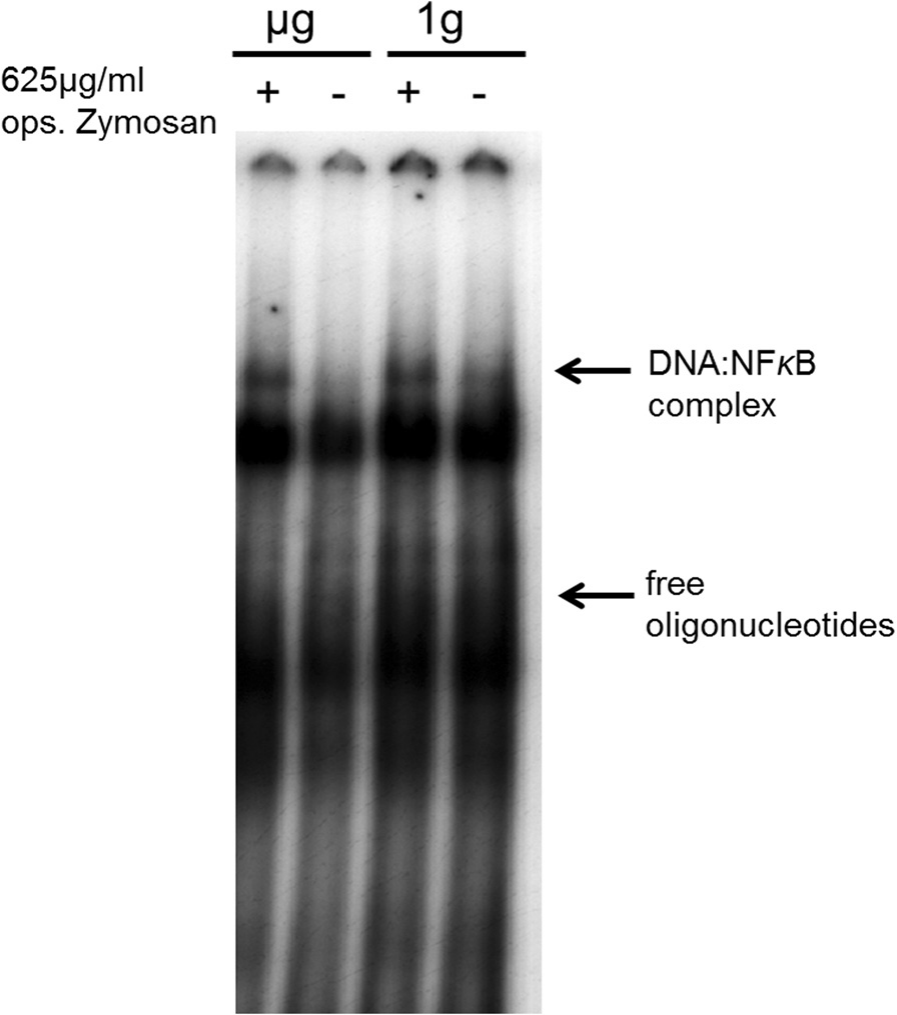
**Radiographic EMSA dried gel, labelled with an NF-κB recognition sequence.** NR8383 cells were stimulated with opsonized zymosan and exposed to clinorotation (Pipette-Clinostat) for 4 h. Nuclear lysates were incubated with ^32^P labelled NF-κB recognition sequences and unravelled upon its mass. Free oligonucleotides run faster compared to NF-κB:DNA complexes. The film represents one of four individual experiments.

The EMSA shows binding of the transcription factor NF-κB to DNA, a process which initiates the transcription of DNA and the production of proteins to modulate the immune response after pathogen recognition. Macrophages were stimulated with opsonized zymosan and exposed to 4 h of clinorotation. Subsequent autoradiography of the EMSA showed no differences in the migration of DNA/NF-κB complexes under conditions of 1 *g* and μ*g* (simulated microgravity) (Figure 6). The results indicate that after exposure to clinorotation, the translocation of NF-κB remains unchanged.

Paulsen et al. [32] reported that, in the monocytic cell line U937, overall tyrosine phosphorylation was reduced during 5 min of clinorotation following stimulation with PMA, although NF-κB (p65) translocation to the nucleus was not affected, which is in line with our data. We can assume that the macrophage cell line used here (NR8383) responds similarly to monocytes, which are the precursors of circulating macrophages.

We can propose that either the NF-κB activation is completely unaffected or the cells adapt to the simulated microgravity conditions, thus fast and early steps (e.g. ROS production and Syk phosphorylation) are highly affected whereas downstream signalling remains unchanged. Adherent melanoma cells show disorder in terms of cell shape but are able to adapt to a lack of gravity [33]. It follows that early signalling might be profoundly impaired but later steps may be still functional due to unknown adaptation processes.

However, NF-κB activation could also occur through a different pathway independent of Syk phosphorylation: Gringhuis et al. [34] showed that NF-κB activation through Dectin-1 receptor stimulation in dendritic cells is mediated, besides the Syk pathway, by the serine-threonine kinase Raf-1, a Syk antagonist.

## Conclusion

The work presented here complements the study of Adrian et al. [19] in terms of shedding light on the signalling steps underlying the oxidative burst of macrophages under altered gravity conditions. The use of different pathogen patterns (non-opsonized zymosan, LPS and curdlan) showed the same effect: the production of oxygen radicals during the oxidative burst is significantly diminished under microgravity conditions. This clearly demonstrates that macrophages are highly susceptible to microgravity conditions and thus distinct signalling pathways are involved. We further addressed signalling during the oxidative burst and concentrated on Syk kinase, which has diverse biological functions [10]. The activation and therefore phosphorylation of this kinase is an early signalling step and a common feature of the pathways involved in initiating the oxidative burst. These results clearly show that microgravity has an effect on the phosphorylation of Syk, which may be a reason for the fast and reversible decrease in ROS in the absence of gravity. However, NF-κB signalling was not influenced by microgravity, which is in line with a study by Gross et al. [12], indicating the adaptation capacity of some cell types.

The mechanism of gravity sensing in single cells is under discussion. The results gained in this study are in favour of the model postulated by Ingber [35-37], since the observed changes in signalling might be the result of altered mechanotransduction. Linker proteins between integrins and the cytoskeleton are candidates which may be highly susceptible to altered force/gravity conditions. Syk is a protein with connections to integrins (via the complement receptor CR3/CD18; [38,39]) and the cytoskeleton [10,40] and shows sensitivity to the absence of gravity. Understanding general force transmittance, and therefore gravisensing in single cells could provide further insight into how spaceflight affects human health. Since different types of cells are differentially affected by the lack of gravity, it can be assumed that the mechanism leading to impaired signalling must be a phenomenon that different cell types have in common.

## Abbreviations

μ*g*: Microgravity
BCA: Bicinchoninic acid assay
CR1/CR3: Complement receptor 1/3
DLR: Deutsches Zentrum für Luft- und Raumfahrt (German Aerospace Center)
EMSA: Electrophoretic mobility shift assay
ESA: European Space Agency
FCS: Foetal calf serum
g: Gram
*g*: Gravity (9.81 m/s^2^)
HRP: Horseradish peroxidase
IκB: Inhibitor of κB
ISS: International Space Station
ITAM: Immunoreceptor tyrosine-based activation motif
LPS: Lipopolysaccharide
NADPH: Nicotinamide adenine dinucleotide phosphate
NF-κB: Nuclear factor kappa-light-chain-enhancer of activated B cells
PBS: Phosphate buffered saline
PRR: Pattern recognition receptor
RLU: Relative light unit
rpm: Revolutions per minute
s: Second
SDS: Sodium dodecyl sulphate
TBST: Tris-buffered saline tween-20
TLR: Toll-like receptor
VLE: Very low endotoxin
